# Association between alcohol consumption and incidence of type 2 diabetes mellitus in Japanese men: a secondary analysis of a Retrospective Cohort Study

**DOI:** 10.1186/s12902-023-01350-1

**Published:** 2023-04-25

**Authors:** Jing Song, Wei-Qian Lin

**Affiliations:** Department of Cardiology, the Second Affiliated Hospital, Yuying Children’s Hospital of Wenzhou Medical University, Wenzhou, 325000 Zhejiang China

**Keywords:** Alcohol consumption, Diabetes mellitus, Cohort study, Japanese

## Abstract

**Background:**

Alcohol consumption is known to be associated with an increased risk of type 2 diabetes (T2DM). However, the effect of alcohol intake on the incidence of T2DM remains controversial due to inconsistent results across studies. This study aimed to bridge the gap among available literature in order to better define the association between alcohol consumption and incidence of T2DM.

**Methods:**

We performed a secondary analysis using open-access data from a retrospective Japanese cohort of 15,464 participants who underwent regular medical examinations at Murakami Memorial Hospital. All participants underwent an initial exam including a questionnaire survey, physical examination, and blood biochemical testing to establish a at baseline. The primary outcome was new-onset T2DM during the follow-up exam. Statistical analysis was conducted using Cox regression and Kaplan–Meier methods to assess the risk of alcohol consumption on T2DM.

**Results:**

During a median follow-up time of 5.39 years, 373 new-onset T2DM events were observed. The cumulative risk of T2DM incidence was higher in the heavy alcohol consumption group vs. the other three groups: none/minimal, light, and moderate consumption (log-rank test, *P* = 0.0002). Multivariate Cox regression analysis indicated incidental T2DM was independently associated with alcohol consumption. The adjusted hazard ratio relative to the none/minimal consumption group was as follows: 1.02 (95% confidence interval: 0.71, 1.48) for light consumption, 1.06 (0.71, 1.57) for moderate consumption, and 2.06 (1.30, 3.24) for heavy consumption (*P* value = 0.024). Subsequent subgroup analysis confirmed the association between alcohol consumption and T2DM incidence in men, but not in women.

**Conclusion:**

Heavy alcohol consumption was independently associated with an increased risk of new-onset T2DM in Japanese men.

## Introduction

Type 2 diabetes mellitus (T2DM) is one of the leading global causes of morbidity and mortality. According to the International Diabetes Federation, 537 million adults have been clinically diagnosed with T2DM; this number is expected to increase to 643 million by 2030 [1].

Increased alcohol intake has been shown to be positively correlated with an increased incidence of T2DM [2]. Comparatively, moderate alcohol consumption has been shown to be associated with a reduced incidence of T2DM [3]. However, the aforementioned results are not consistent among other studies [4–6]. These discrepancies may reflect differences in analytical methodology used to examine the association, ethnic, and/or lifestyle differences among the study populations. In addition, some of the previous studies included participants who had preexisting diabetes whereas other studies included biased populations. The NAGALA (NAfld in the Gifu Area, Longitudinal Analysis) study was conducted at Murakami Memorial Hospital’s Medical Health Checkup Center (Gifu, Japan) from 2004 to 2015; key advantages of the NAGALA study are the large sample size and rigorous follow-up over an extended period of time.

The current study describes a secondary analysis of the NAGALA study to examine the relationship between alcohol consumption and incidence of T2DM.

## Methods

### Data source

Data was obtained from the publicly forum “DRYAD” (https://datadryad.org/) and from the following article: Ectopic fat obesity presents the greatest risk for incident T2DM: a population-based longitudinal study [7] (dataset: 10.5061/dryad.8q0p192). The database file contains the following variables: age, sex, body weight, body mass index (BMI), waist circumference, systolic blood pressure (SBP), diastolic blood pressure (DBP), alanine aminotransferase (ALT), aspartate aminotransferase (AST), gamma-glutamyltransferase (GGT), total cholesterol (TC), triglyceride (TG), high-density lipoprotein cholesterol (HDL-C), fasting plasma glucose (FPG), hemoglobin A1C (HbA1C), alcohol consumption, exercise, smoking status, fatty liver, year of follow-up and T2DM events during the follow-up. The authors of the original study forfeited all copyrights and related ownership of the data used in this secondary analysis. The original study was approved by the Murakami Memorial Hospital Ethics Committee. Written informed consent was obtained from all participants. The authors state that this study was conducted in accordance with the Declaration of Helsinki.

### Study design and participants

The NAGALA study was conducted at Murakami Memorial Hospital’s Medical Health Checkup Center (Gifu, Japan). A total of 20,944 participants were recruited during a period from 2004 to 2015. The final analysis excluded participants with missing covariate data, known liver disease, excessively heavy alcohol intake (ethanol consumption over 60 g/day for men and 40 g/day for women), medication use, and diagnosis of T2DM or FPG ≥ 6.1 mmol/L. Ultimately, 15,464 participants were included in the original study [7].

### Data collection and measurements

The following information was obtained at the initial screening: weight, waist circumference, blood pressure and a standard blood test panel that included alanine aminotransferase (ALT), aspartate aminotransferase (AST), gamma-glutamyltransferase (GGT), total cholesterol (TC), triglyceride (TG), high-density lipoprotein cholesterol (HDL-C), fasting plasma glucose (FPG), and hemoglobinA1c (HbA1c) levels in fasting blood samples. Medical history and lifestyle variables, such as smoking and drinking habits, were collected using a standardized questionnaire. Alcohol consumption estimation was based on the mean weekly alcohol intake, as assessed by asking the participants about the type and amount of alcohol consumed each week during the prior month, and classified into four categories according to the original study: heavy = > 280 g per week; moderate = 140–280 g per week; light = 40–140 g per week; and none or minimal = < 40 g per week [7, 8]. Smoking status was classified into three categories: never smoking, past smoking, and currently smoking [7]. Regular physical activity was defined as engaging in any sporting activity (exercise) more than once per week [9]. Abdominal ultrasound was performed by a trained technician, and images were reviewed by a gastroenterologist blinded to other personal data of the participants. The diagnosis of fatty liver disease was based on ultrasound images and classified into four criteria: hepatorenal echo contrast, liver brightness, deep attenuation, and vascular blurring [7]. An estimated 60% of the participants were examined once or twice per year [7]. T2DM was defined as FPG ≥ 7.0 mmol/L, HbA1c ≥ 6.5%, or self-reported. The study endpoint was the occurrence of T2DM during the follow-up.

### Statistical analysis

Continuous variables were compared among groups with different alcohol consumption categories using one-way ANOVA, Kruskal-Wallis H test, and shown as mean ± standard deviation (SD) or median and quaternary ranges (25th-75th percentile) based on data distribution (normal vs. skewed). Categorical variables were compared using chi-square test, and shown as number and percentages. Cumulative incidence of T2DM was plotted using Kaplan–Meier curves and analyzed using a log-rank test. Multivariate Cox regression was used to evaluate the potential associations between alcohol consumption and T2DM risk. Results are shown as the hazard ratio (HR) and 95% confidence interval (95%CI). Adjustments were made when HR changed by 10% upon covariate addition. Smooth curve fitting was conducted for visual display of the relationship between alcohol consumption and new-onset T2DM.

All analyses were performed with the statistical software package R (http://www.R-project.org, The R Foundation) and Empower-Stats (http://www.empowerstats.com, X&Y Solutions, Inc., Boston, MA). All T tests were two-sided, and P values < 0.05 were considered statistically significant.

## Results

### Baseline characteristics of the Study Population

The final analysis included a total of 15,464 participants (8430 men and 7034 women). The average age was 43.71 ± 8.90 years (Table 1).

**Table 1:** Baseline characteristics of participants classified according to alcohol consumption

	None or minimal	Light	Moderate	Heavy	*P*
	N = 11,805	N = 1,758	N = 1,360	N = 541	
Age (y)	43.17 ± 8.86	44.73 ± 8.72	45.89 ± 8.96	46.63 ± 8.47	< 0.001
Male, sex	5354 (45.35%)	1369 (77.87%)	1166 (85.74%)	541 (100.00%)	< 0.001
BMI (kg/m^2^)	21.96 ± 3.18	22.42 ± 2.94	22.66 ± 2.82	23.10 ± 2.78	< 0.001
Waist circumference (cm)	75.59 ± 9.18	78.43 ± 8.34	79.52 ± 8.26	81.69 ± 7.40	< 0.001
Smoking status					< 0.001
Never	7907 (66.98%)	681 (38.74%)	350 (25.74%)	93 (17.19%)	
Past	1812 (15.35%)	515 (29.30%)	448 (32.94%)	177 (32.72%)	
Current	2086 (17.67%)	562 (31.97%)	562 (41.32%)	271 (50.09%)	
Exercise					< 0.001
No	9832 (83.29%)	1398 (79.52%)	1094 (80.44%)	431 (79.67%)	
Yes	1973 (16.71%)	360 (20.48%)	266 (19.56%)	110 (20.33%)	
Fatty liver disease					0.035
No	9717 (82.31%)	1472 (83.73%)	1110 (81.62%)	424 (78.37%)	
Yes	2088 (17.69%)	286 (16.27%)	250 (18.38%)	117 (21.63%)	
ALT (IU/L)	16.00 (12.00, 22.00)	18.00 (14.00, 25.00)	19.00 (15.00, 25.00)	21.00 (17.00, 29.00)	< 0.001
AST (IU/L)	17.00 (14.00, 21.00)	18.00 (14.00, 21.00)	19.00 (15.00, 23.00)	20.00 (16.00, 25.00)	< 0.001
GGT (IU/L)	14.00 (11.00–19.00)	19.00 (14.00–28.00)	23.00 (16.00–35.00)	30.00 (21.00–46.00)	< 0.001
HDL-C (mmol/L)	1.47 ± 0.40	1.43 ± 0.41	1.46 ± 0.42	1.43 ± 0.41	0.002
TC (mmol/L)	5.12 ± 0.87	5.09 ± 0.84	5.16 ± 0.84	5.18 ± 0.79	0.091
TG (mmol/L)	0.70 (0.46, 1.06)	0.80 (0.56, 1.22)	0.89 (0.61, 1.36)	1.03 (0.70, 1.60)	< 0.001
HbA1C (%)	5.19 ± 0.32	5.12 ± 0.32	5.11 ± 0.32	5.09 ± 0.34	< 0.001
FPG (mmol/L)	5.12 ± 0.41	5.26 ± 0.40	5.31 ± 0.39	5.37 ± 0.37	< 0.001
SBP (mmHg)	113.08 ± 14.64	117.27 ± 15.19	119.76 ± 14.73	123.13 ± 15.08	< 0.001
DBP (mmHg)	70.41 ± 10.24	73.94 ± 10.38	75.82 ± 10.31	78.78 ± 10.16	< 0.001

**Note**: Data are expressed as n (%) or mean ± SD or medians (interquartile range:25th to 75th percentiles)

**Abbreviations**: BMI, body mass index; AST, aspartate aminotransferase; ALT, alanine aminotransferase; GGT, gamma-glutamyltransferase; HDL-C, high-density lipoprotein cholesterol; TC, total cholesterol; TG, triglyceride; FPG, fasting plasma glucose; HbA1c, hemoglobinA1c; SBP, systolic blood pressure; DBP, diastolic blood pressure

The percentages of the study cohorts with respect to different alcohol consumption categories were: 76.34% (11,805) for none or minimal, 11.37% (1758) for light, 8.80% (1360) for moderate and 3.50% (541) for heavy alcohol consumption. As the categorization of alcohol consumption was constrained by the original study, the heavy drink consumption in women could not be analysed, there were no women in the heavy consumption group. Overall, participants in the heavy consumption group were of older age comparatively, had higher BMI and waist circumference, higher blood pressure, and higher levels of ALT, AST, GGT, TG and FPG, but lower HbA1C. The rate of fatty liver disease of those currently smoking and the current smoker was higher in the heavy consumption group.

**Fig. 1:**
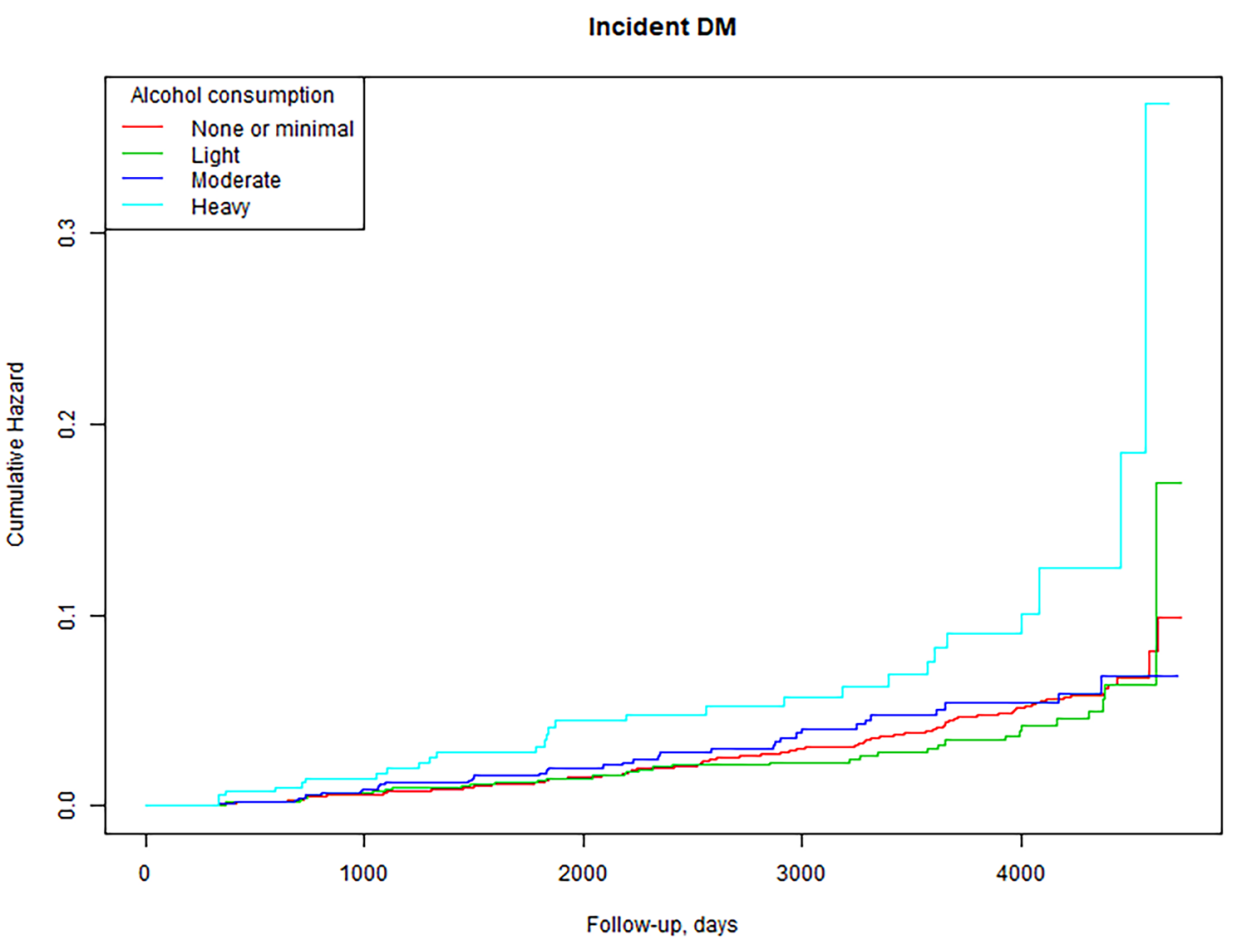
Cumulative hazard curves for incidental T2DM by alcohol consumption

Kaplan–Meier analysis revealed higher cumulative risk of T2DM in heavy consumption group compared to all other groups (log rank test, *P* = 0.0002; Fig. 1).

### Cox regression analysis

Univariate regression analysis revealed incidental T2DM was positively associated with age, BMI, waist circumference, ALT, AST, GGT, TG, FPG, HbA1C, SBP, DBP, current smokers, fatty liver disease and heavy alcohol consumption, and negatively with HDL-C (Table 2).

**Table 2:** Univariate analysis between variables and incident diabetes

	Statistics	HR (95% CI)	*P*-value
Age	43.71 ± 8.90	1.06 (1.04, 1.07)	< 0.00001
Male	8430 (54.51%)	2.52 (1.98, 3.21)	< 0.00001
BMI	22.12 ± 3.13	1.24 (1.22, 1.27)	< 0.00001
Waist circumference	76.48 ± 9.11	1.09 (1.08, 1.10)	< 0.00001
Smoking status			
Never	9031 (58.40%)	1.0	
Past	2952 (19.09%)	1.66 (1.26, 2.18)	0.00035
Current	3481 (22.51%)	2.58 (2.06, 3.24)	< 0.00001
Exercise			
No	12,755 (82.48%)	1.0	
Yes	2709 (17.52%)	0.76 (0.56, 1.02)	0.06410
Fatty liver disease			
No	12,723 (82.28%)	1.0	
Yes	2741 (17.73%)	7.02 (5.70, 8.63)	< 0.00001
Alcohol consumption			
None or minimal	11,805 (76.34%)	1.0	
Light	1758 (11.37%)	0.90 (0.65, 1.26)	0.55084
Moderate	1360 (8.80%)	1.15 (0.82, 1.62)	0.42404
Heavy	541 (3.50%)	2.24 (1.54, 3.27)	0.00003
ALT	19.99 ± 14.34	1.01 (1.01, 1.01)	< 0.00001
AST	18.40 ± 8.64	1.01 (1.01, 1.01)	< 0.00001
GGT	20.31 ± 18.14	1.01 (1.01, 1.01)	< 0.00001
HDL-C	1.46 ± 0.40	0.15 (0.11, 0.20)	< 0.00001
TC	5.126 ± 0.864	1.49 (1.34, 1.66)	< 0.00001
TG	0.912 ± 0.655	1.80 (1.68, 1.92)	< 0.00001
HbA1C	5.172 ± 0.322	54.27 (39.49, 74.59)	< 0.00001
FPG	5.16 ± 0.41	25.38 (18.71, 34.42)	< 0.00001
SBP	114.50 ± 14.97	1.03 (1.03, 1.04)	< 0.00001
DBP	71.58 ± 10.51	1.05 (1.04, 1.06)	< 0.00001

Multivariate regression analysis revealed incidental T2DM was independently associated with older age, higher BMI, SBP, ALT, GGT, HDL-C, TC, TG, FPG, HbA1C, fatty liver disease and smoking status, as well as alcohol consumption (Table 3). The adjusted HR relative to the none or minimal consumption group was 1.02 (0.71, 1.48) for light consumption, 1.06 (0.71, 1.57) for moderate consumption, and 2.06 (1.30, 3.24) for heavy consumption (*P* value = 0.024). The association between heavy consumption with incidental T2DM was significantly different in the subgroup analysis that only included men (HR: 2.06, 95%CI: 1.30–3.28; *P value* = 0.002), but not in women.

**Table 3:** Relationship between alcohol consumption and incident diabetes in different models

	Model 1 (HR, 95% CI, *P*)	Model 2 (HR, 95% CI, *P*)	Model 3 (HR, 95% CI, *P*)
**Total**			
Alcohol consumption			
None or minimal	Ref	Ref	Ref
Light	0.76 (0.54, 1.07) 0.116	0.71 (0.51, 1.00) 0.052	1.02 (0.71, 1.48) 0.907
Moderate	0.86 (0.61, 1.23) 0.414	0.77 (0.54, 1.10) 0.150	1.06 (0.71, 1.57) 0.782
Heavy	1.64 (1.11, 2.44) 0.014	1.45 (0.97, 2.16) 0.068	2.06 (1.30, 3.24) 0.002
*P* for trend	0.394	0.973	0.024
**Sex = male**			
Alcohol consumption			
None or minimal	Ref	Ref	Ref
Light	0.80 (0.56, 1.14) 0.223	0.75 (0.53, 1.07) 0.116	1.07 (0.72, 1.58) 0.736
Moderate	0.84 (0.58, 1.22) 0.371	0.76 (0.52, 1.10) 0.146	1.03 (0.68, 1.57) 0.876
Heavy	1.65 (1.11, 2.45) 0.013	1.47 (0.99, 2.19) 0.058	2.06 (1.30, 3.28) 0.002
*P* for trend	0.328	0.812	0.026
**Sex = female**			
Alcohol consumption			
None or minimal	Ref	Ref	Ref
Light	0.40 (0.10, 1.64) 0.203	0.40 (0.10, 1.61) 0.195	0.60 (0.14, 2.55) 0.487
Moderate	1.22 (0.38, 3.90) 0.737	1.18 (0.37, 3.78) 0.780	1.32 (0.34, 5.05) 0.687
Heavy	-	-	-
*P* for trend	0.658	0.619	0.998

Model 1, unadjusted; model 2, adjusted for age; and model 3, adjusted terms for age, BMI, SBP, ALT, GGT, HDL-C, TC, TG, FPG, HbA1C, fatty liver disease and smoking status

### Nonlinear relationship between Alcohol Consumption and T2DM

**Fig. 2:**
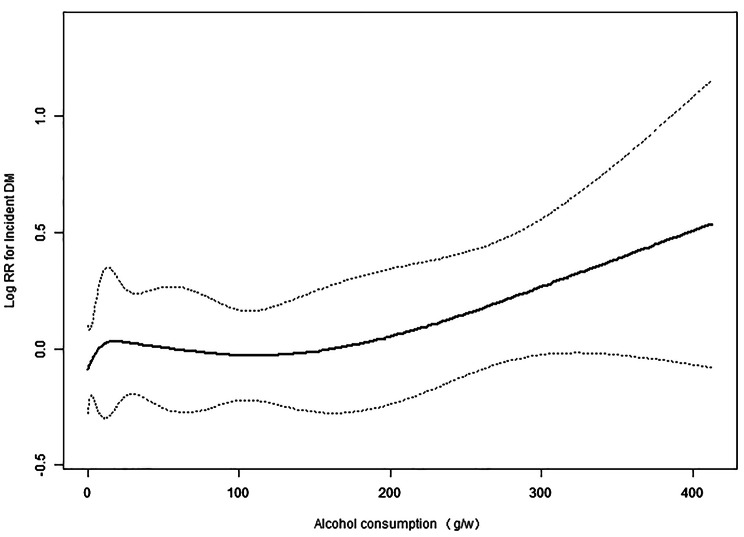
The association between alcohol consumption and T2DM events in men, after adjustment for age, BMI, SBP, ALT, GGT, HDL-C, TC, TG, FPG, HbA1C, fatty liver disease and smoking status

The nonlinear relationship between alcohol consumption and T2DM risk, after adjusting for confounding factors (e.g., age, BMI, SBP, ALT, GGT, HDL-C, TC, TG, FPG, HbA1C, fatty liver disease and smoking status) in men, is shown in Fig. 2. There were no saturation or threshold effects (*P* = 0.385).

## Discussion

In the present study, T2DM incidence was independently associated with heavy alcohol consumption. The adjusted HR relative to the none or minimal consumption group was 1.02 (0.76, 1.48) for light consumption, 1.06 (0.71, 1.57) for moderate consumption, and 2.06 (1.30, 3.24) for heavy consumption (*P* value = 0.024). Subgroup analysis confirmed the association between alcohol consumption and incidental T2DM in men but not in women. The subgroup analysis in men suggested a nonlinear relationship between alcohol intake and incidental T2DM; however, this relationship was not statistically significant.

Heavy alcohol consumption has been consistently associated with incident T2DM [10, 11]. For instance, Watanabe et al. [12] found that alcohol intake increased the probability of developing T2DM in Japanese men. In a study by Cullmann et al. [2], men with heavy alcohol consumption are more likely to have impaired glucose regulation. Moderate alcohol consumption has been associated with reduced risk of T2DM [13–15]. However, in some Asian groups, this protective effect on T2DM incidence was not found [16, 17]. In the current study, we did not find a reduced risk of incidental T2DM with moderate alcohol consumption. Similar discrepancies are reflected among ethnic backgrounds throughout associated literature.

There are several biological mechanisms that might explain the reported association between alcohol consumption and T2DM. Heavy alcohol drinking causes diabetes by increasing caloric intake and obesity, which disrupts carbohydrate and glucose metabolism. Heavy alcohol consumption has also been linked to chronic pancreatitis and liver injury, with decreased insulin secretion and insulin resistance [18, 19]. Previous research has associated disruption of β-cells to oxidative stress, mitochondrial dysfunction, and increased generation of reactive oxygen species in pancreatic islets [2, 15]. In vitro, ethanol metabolites 2,3-butanediol and 1,2-propanediol inhibit both basal and insulin-stimulated adipocyte metabolism [20]. Ethanol exposure in rats increases β-cell atrophy and decreases absolute pancreatic islet volume [21]. β-cells from ethanol-treated rats had extensive ultrastructural abnormalities, including deep invagination of the nuclear envelope, heterochromatin margination, and numerous empty granules or granules lacking distinct electrodense insulin crystals. Wang et al. [22] showed that excessive ethanol consumption disrupted pancreatic γ-aminobutyric acid (GABA) signaling in pancreatic islet β-cells and compromised glucose metabolism. The mechanisms underlying impaired pancreatic islet glucose metabolism and insulin secretion capacity are complex, and may involve resistance to fibroblast growth factor 21 (FGF21) [23]. A recent study linked increased T2DM by alcohol consumption to a HECTD4 genotype [24]. Specifically, HECTD4 mutation predisposes people to T2DM upon alcohol consumption.

Previous studies have also suggested a J- or U-shaped relationship between alcohol consumption and incidental diabetes [25, 26]. Subgroup analysis in the current study confirmed an independent association between alcohol consumption and incidental diabetes in men but not women. The subgroup analysis in men suggested a nonlinear relationship, albeit statistically non-significant, between alcohol consumption and incident T2DM. Several previous studies in the Western population showed that moderate alcohol consumption is associated with an increase in HDL-C, which in turn reduces the risk of incident T2DM [27, 28]. The current study failed to show reduced risk of incident T2DM with moderate alcohol consumption. The observed discrepancy may be partly attributed to different ethnic backgrounds; metabolic differences between Japanese and Western populations have been documented previously. For example, Western populations have higher levels of insulin resistance whereas Japanese populations are less likely to be obese yet have greater β-cell dysfunction [29]. Accordingly, the effect of heavy alcohol intake on incidental T2DM in Japanese men may be explained by its influence on β-cell function rather in insulin sensitivity.

A major strength of the current study is the large sample size and rigorous follow-up for an extended period of time in the original study. As a population-based retrospective cohort study, the data on alcohol consumption and incident diabetes, as well as a broad range of confounding factors, are relatively reliable.

The current study has several limitations. First, as a secondary analysis, we did not have access to all confounding factors. Second, the diagnosis of incidental T2DM was not based on an oral glucose tolerance test and thus may be underestimated. Third, alcohol consumption status was based on the information collected at baseline did not consider changes during the follow-up period. Fourth, according to the categorization of alcohol in women, the number of women with moderate alcohol consumptions was very low; heavy drinking was not reported by any women in this cohort.

In conclusion, the current study revealed an independent association between heavy alcohol consumption and increased risk of incidental T2DM. Subgroup analysis confirmed the association in men, but not in women.

## Data Availability

The datasets used and/or analyzed during the current study are available from “DRYAD” database (dataset: 10.5061/dryad.8q0p192).

## Abbreviations

ALT: Alanine aminotransferase
AST: Aspartate aminotransferase
BMI: Body mass index
DBP: Diastolic blood pressure
FPG: Fasting plasma glucose
GGT: Gamma-glutamyltransferase
HbA1c: HemoglobinA1c
HDL-C: High-density lipoprotein cholesterol
SBP: Systolic blood pressure
TC: Total cholesterol
TG: Triglyceride

## References

[1] Saeedi P, Petersohn I, Salpea P, Malanda B, Karuranga S, Unwin N, Colagiuri S, Guariguata L, Motala AA, Ogurtsova K (2019). Global and regional diabetes prevalence estimates for 2019 and projections for 2030 and 2045: results from the International Diabetes Federation Diabetes Atlas, 9(th) edition. Diabetes Res Clin Pract.

[2] Cullmann M, Hilding A, Ostenson CG (2012). Alcohol consumption and risk of pre-diabetes and type 2 diabetes development in a swedish population. Diabet Med.

[3] Carlsson S, Hammar N, Grill V (2005). Alcohol consumption and type 2 diabetes Meta-analysis of epidemiological studies indicates a U-shaped relationship. Diabetologia.

[4] Zilkens RR, Puddey IB (2003). Alcohol and cardiovascular disease–more than one paradox to consider. Alcohol and type 2 diabetes–another paradox?. J Cardiovasc Risk.

[5] Saremi A, Hanson RL, Tulloch-Reid M, Williams DE, Knowler WC (2004). Alcohol consumption predicts hypertension but not diabetes. J Stud Alcohol.

[6] Li XH, Yu FF, Zhou YH, He J (2016). Association between alcohol consumption and the risk of incident type 2 diabetes: a systematic review and dose-response meta-analysis. Am J Clin Nutr.

[7] Okamura T, Hashimoto Y, Hamaguchi M, Obora A, Kojima T, Fukui M (2019). Ectopic fat obesity presents the greatest risk for incident type 2 diabetes: a population-based longitudinal study. Int J Obes (Lond).

[8] Hashimoto Y, Hamaguchi M, Kojima T, Ohshima Y, Ohbora A, Kato T, Nakamura N, Fukui M (2015). Modest alcohol consumption reduces the incidence of fatty liver in men: a population-based large-scale cohort study. J Gastroenterol Hepatol.

[9] Ryu S, Chang Y, Kim DI, Kim WS, Suh BS (2007). Gamma-glutamyltransferase as a predictor of chronic kidney disease in nonhypertensive and nondiabetic korean men. Clin Chem.

[10] Roh WG, Shin HC, Choi JH, Lee YJ, Kim K (2009). Alcohol consumption and higher incidence of impaired fasting glucose or type 2 diabetes in obese korean men. Alcohol.

[11] Tsumura K, Hayashi T, Suematsu C, Endo G, Fujii S, Okada K (1999). Daily alcohol consumption and the risk of type 2 diabetes in japanese men: the Osaka Health Survey. Diabetes Care.

[12] Watanabe M, Barzi F, Neal B, Ueshima H, Miyoshi Y, Okayama A, Choudhury SR (2002). Alcohol consumption and the risk of diabetes by body mass index levels in a cohort of 5,636 japanese. Diabetes Res Clin Pract.

[13] Zhang S, Liu Y, Wang G, Xiao X, Gang X, Li F, Sun C, Gao Y. The relationship between alcohol consumption and incidence of glycometabolic abnormality in middle-aged and elderly Chinese men. *Int J Endocrinol* 2016; 2016:1983702. 10.1155/2016/1983702 PMC4769752 26981121

[14] Baliunas DO, Taylor BJ, Irving H, Roerecke M, Patra J, Mohapatra S, Rehm J (2009). Alcohol as a risk factor for type 2 diabetes: a systematic review and meta-analysis. Diabetes Care.

[15] Ajani UA, Hennekens CH, Spelsberg A, Manson JE (2000). Alcohol consumption and risk of type 2 diabetes mellitus among US male physicians. Arch Intern Med.

[16] Knott C, Bell S, Britton A (2015). Alcohol consumption and the risk of type 2 diabetes: a systematic review and dose-response meta-analysis of more than 1.9 million individuals from 38 observational studies. Diabetes Care.

[17] Park SY, Jeong SJ, Ustulin M, Chon S, Woo JT, Lim JE, Oh B, Rhee SY (2019). Incidence of diabetes mellitus in male moderate alcohol drinkers: a community-based prospective cohort study. Arch Med Res.

[18] Nealon WH, Townsend CM, Thompson JC (1988). The time course of beta cell dysfunction in chronic ethanol-induced pancreatitis: a prospective analysis. Surgery.

[19] Ikai E, Ishizaki M, Suzuki Y, Ishida M, Noborizaka Y, Yamada Y (1995). Association between hepatic steatosis, insulin resistance and hyperinsulinaemia as related to hypertension in alcohol consumers and obese people. J Hum Hypertens.

[20] Lomeo F, Khokher MA, Dandona P (1988). Ethanol and its novel metabolites inhibit insulin action on adipocytes. Diabetes.

[21] Koko V, Todorovic V, Nikolic JA, Glisic R, Cakic M, Lackovic V, Petronijevic L, Stojkovic M, Varagic J, Janic B (1995). Rat pancreatic B-cells after chronic alcohol feeding. A morphometric and fine structural study. Histol Histopathol.

[22] Wang S, Luo Y, Feng A, Li T, Yang X, Nofech-Mozes R, Yu M, Wang C, Li Z, Yi F (2014). Ethanol induced impairment of glucose metabolism involves alterations of GABAergic signaling in pancreatic beta-cells. Toxicology.

[23] Yang BC, Wu SY, Leung PS (2020). Alcohol ingestion induces pancreatic islet dysfunction and apoptosis via mediation of FGF21 resistance. Ann Transl Med.

[24] Lee YJ, Lee H, Jang HB, Yoo MG, Im S, Koo SK, Lee HJ (2022). The potential effects of HECTD4 variants on fasting glucose and triglyceride levels in relation to prevalence of type 2 diabetes based on alcohol intake. Arch Toxicol.

[25] Wu X, Liu X, Liao W, Kang N, Dong X, Abdulai T, Zhai Z, Wang C, Wang X, Li Y (2021). Prevalence and characteristics of alcohol consumption and risk of type 2 diabetes mellitus in rural China. BMC Public Health.

[26] Lee DY, Yoo MG, Kim HJ, Jang HB, Kim JH, Lee HJ, Park SI (2017). Association between alcohol consumption pattern and the incidence risk of type 2 diabetes in korean men: a 12-years follow-up study. Sci Rep.

[27] Sierksma A, van der Gaag MS, van Tol A, James RW, Hendriks HF (2002). Kinetics of HDL cholesterol and paraoxonase activity in moderate alcohol consumers. Alcohol Clin Exp Res.

[28] Rimm EB, Williams P, Fosher K, Criqui M, Stampfer MJ (1999). Moderate alcohol intake and lower risk of coronary heart disease: meta-analysis of effects on lipids and haemostatic factors. BMJ.

[29] Yabe D, Seino Y, Fukushima M, Seino S (2015). beta cell dysfunction versus insulin resistance in the pathogenesis of type 2 diabetes in East Asians. Curr Diab Rep.

